# Crizotinib acts as ABL1 inhibitor combining ATP-binding with allosteric inhibition and is active against native BCR-ABL1 and its resistance and compound mutants BCR-ABL1^T315I^ and BCR-ABL1^T315I-E255K^

**DOI:** 10.1007/s00277-020-04357-z

**Published:** 2021-06-10

**Authors:** Afsar Ali Mian, Isabella Haberbosch, Hazem Khamaisie, Abed Agbarya, Larissa Pietsch, Elizabeh Eshel, Dally Najib, Claudia Chiriches, Oliver Gerhard Ottmann, Oliver Hantschel, Ricardo M. Biondi, Martin Ruthardt, Jamal Mahajna

**Affiliations:** 1 Department of Hematology, Division of Cancer and Genetics, and Experimental Clinical Medical Center (ECMC), Medical School, Cardiff University, Heath Park, Cardiff, CF14 4XN UK; 2 Center for Regenerative Medicine and Stem Cell Research, Aga Khan University, Karachi, Pakistan; 3 Department of Internal Medicine II, Goethe University, Frankfurt, Germany; 4 Department of Nutrition and Natural Products, Migal-Galilee Technology Center, PO Box 831, 11016 Kiryat Shmona, Israel; 5 Oncology Department, Bnai Zion MC, Haifa, Israel; 6 Department of Internal Medicine I, Clinic of Goethe University, Frankfurt, Germany; 7 German Cancer Consortium (DKTK), Frankfurt, Germany; 8 Hematology Institute, Ziv Medical Center, Azrieli Faculty of Medicine, Bar Ilan University, Zfat, Israel; 9 Swiss Institute for Experimental Cancer Research, School of Life Sciences, École polytechnique fédérale de Lausanne, Lausanne, Switzerland; 10 Medical Biochemistry and Pharmacology Center, Institute for Physiological Chemistry, Philipps-University, Marburg, Germany; 11 Instituto de Investigación en Biomedicina de Buenos Aires (IBioBA)-CONICET-Partner Institute of the Max Planck Society, Buenos Aires, Argentina; 12 The Department of Nutritional Sciences, Tel Hai Academic College, Kiryat Shmona, Israel

**Keywords:** Crizotinib, Philadelphia chromosome–positive leukemia, BCR-ABL1, TKI resistance, Allosteric inhibition, Compound mutations

## Abstract

Resistance remains the major clinical challenge for the therapy of Philadelphia chromosome–positive (Ph+) leukemia. With the exception of ponatinib, all approved tyrosine kinase inhibitors (TKIs) are unable to inhibit the common “gatekeeper” mutation T315I. Here we investigated the therapeutic potential of crizotinib, a TKI approved for targeting ALK and ROS1 in non-small cell lung cancer patients, which inhibited also the ABL1 kinase in cell-free systems, for the treatment of advanced and therapy-resistant Ph+ leukemia. By inhibiting the BCR-ABL1 kinase, crizotinib efficiently suppressed growth of Ph+ cells without affecting growth of Ph− cells. It was also active in Ph+ patient-derived long-term cultures (PD-LTCs) independently of the responsiveness/resistance to other TKIs. The efficacy of crizotinib was confirmed in vivo in syngeneic mouse models of BCR-ABL1- or BCR-ABL1^T315I^-driven chronic myeloid leukemia–like disease and in BCR-ABL1-driven acute lymphoblastic leukemia (ALL). Although crizotinib binds to the ATP-binding site, it also allosterically affected the myristol binding pocket, the binding site of GNF2 and asciminib (former ABL001). Therefore, crizotinib has a seemingly unique double mechanism of action, on the ATP-binding site and on the myristoylation binding pocket. These findings strongly suggest the clinical evaluation of crizotinib for the treatment of advanced and therapy-resistant Ph+ leukemia.

## Introduction

Although tyrosine kinase inhibitors (TKIs) directed against BCR-ABL1 have greatly improved the outcome of Philadelphia chromosome–positive (Ph+) leukemia [1, 2], there is still the need of effective and safe agents for treatment of patients with advanced, CML-blast crisis (BC), or Ph+ ALL, where the impact of TKI has been less significant, or therapy-resistant Ph+ leukemia [3]⁠. ln these patients and approximately 50% of imatinib-resistant CML patients overall, second-generation TKIs, such as nilotinib, dasatinib, or bosutinib, did not resolve the problem of resistance. In fact, TKD mutations related to clinical resistance reflect the relative potency of the individual TKIs towards different mutations in the kinase domain [4, 5]. As a consequence, the inferior survival of patients resistant to TKI highlights the need for more effective therapy.

Clinically most challenging is the gatekeeper mutation T315I due to its resistance to all approved second-generation TKIs. Only ponatinib, a multi-kinase inhibitor, which targets potently native BCR-ABL1, BCR-ABL1^T315I^, and all known single BCR-ABL1 mutants in vitro as well as in vivo is approved for treatment of CML and Ph+ ALL patients with multi-TKI-resistant disease or presence of the T315I mutation [6]⁠⁠.

In advanced Ph+ leukemia, responses to TKIs are limited and transient, despite administration guided by mutational analysis. In these cases, mechanisms of resistance are often unknown but include amplification of BCR-ABL1, aberrant phosphatase activity, drug transporter activity, and “sanctuary sites,” e.g., the CNS [7]⁠.

All these therapeutic obstacles including intolerability and adverse side effect emerging with chronic exposition to a given TKI prompted us to explore the therapeutic potential of FDA-approved TKIs with activity against the ABL1 kinase, which revealed crizotinib as one of the most promising candidates. Crizotinib is an inhibitor of mesenchymal-epithelial transition factor (MET)/hepatocyte growth factor receptor (HGFR) and anaplastic lymphoma kinase (ALK) and several other kinases [8]. As crizotinib was previously only shown to potently inhibit ABL1 in vitro but not in Ph+ cells [8]⁠, we studied the effects of clinically feasible concentrations of crizotinib on the kinase activity of BCR-ABL1 and its resistance mutants.

## Results and discussion

**Fig. 1:**
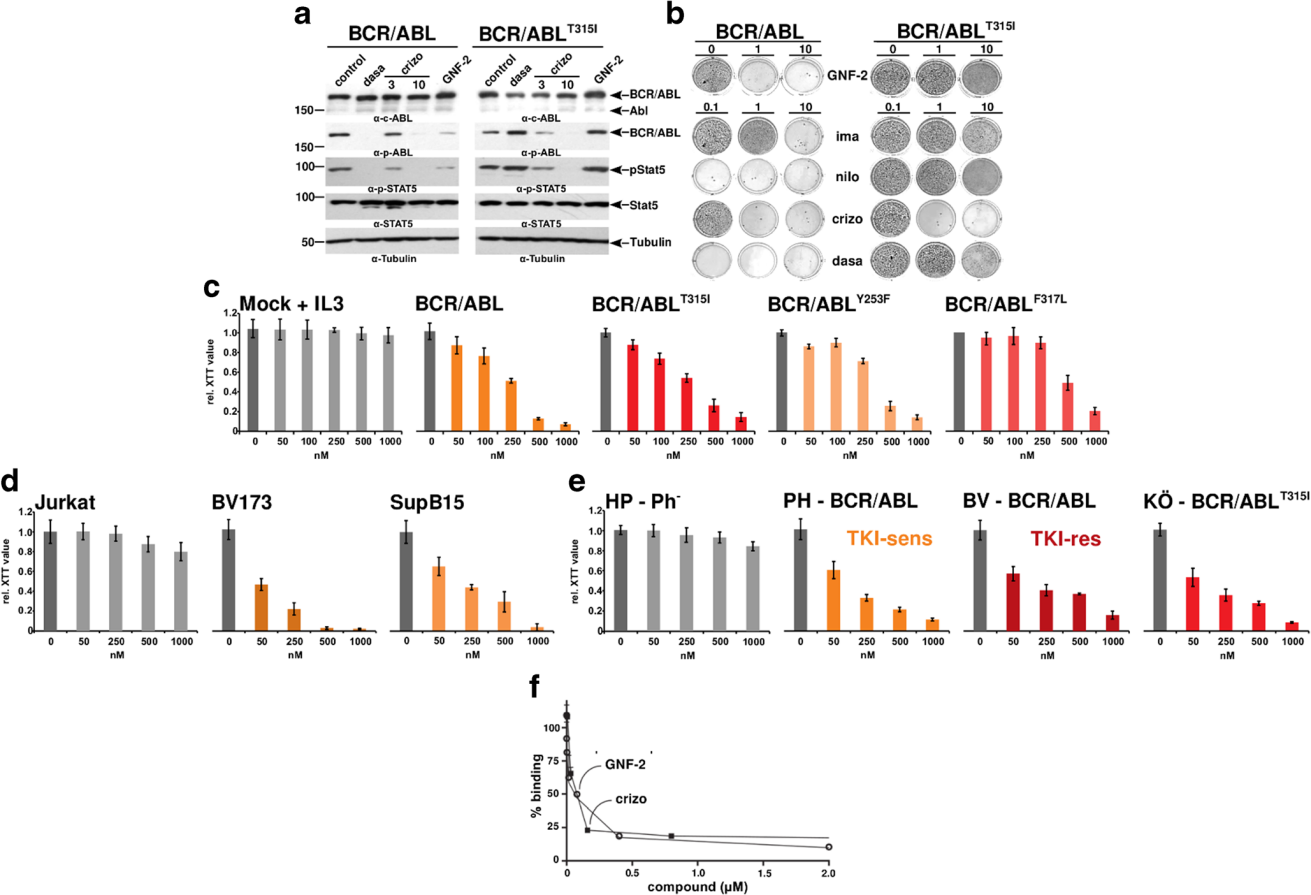
Inhibition of the ABL1 kinase activity by crizotinib blocks the factor-independent growth of Ba/F3 cells mediated by BCR-ABL1 and BCR-ABL1^T315I^. **a** Western blot analysis of lysates of Ba/F3 cells expressing BCR-ABL1 and BCR-ABL1^T315I^ using antibodies directed against the following targets: c-Abl, Abl-Y245 (anti-phospho-ABL1), Crkl, phosphorylated Crkl, Stat5, phosphorylated Stat5 (anti-phospho-STAT5), and β-tubulin (anti-β-tubulin). Molecular mass reference (kDa) values are presented, and c-Abl and β-tubulin were used as loading controls. To avoid bias of stress-induced signaling by factor withdrawal, we performed these experiments in the presence of IL-3. **b** The effect of crizotinib on the factor-independent growth of Ba/F3 cells expressing BCR-ABL1 or BCR-ABL1^T315I^ was assessed in cells selected by IL-3 withdrawal. These cells were seeded in semi-solid medium and exposed to the indicated concentrations of GNF-2 (allosteric inhibitor of ABL1), imatinib, nilotinib, crizotinib, and dasatinib. At day 14, colonies were stained. **c** The effect of crizotinib on cell proliferation and viability of factor-independent Ba/F3 cells upon the expression of BCR-ABL1, BCR-ABL1^T315I^, BCR-ABL1^Y253F^, and BCR-ABL1^F317L^ was assessed with an XTT assay. The lack of cytotoxic effects was confirmed in empty vector-transduced Ba/F3 cells in the presence of IL-3 at a concentration of less than 1 μM. The mean of three experiments ± SD is given. **d** Crizotinib inhibits human Ph+ patient-derived cell lines and primary Ph+ ALL PD-LTCs. Proliferation/cytotoxicity assays using XTT were performed on human Ph+ cell lines derived from Ph+ ALL or CML patients. SupB15 (Ph+ ALL) expressing p185^BCR-ABL1^ or BV-173 cells expressing p210^BCR-ABL1^ (lymphocytic CML-BC cells) were exposed to increasing concentrations of crizotinib. The means of three experiments ± SD each performed in triplicates are given. **e** Proliferation of PD-LTCs - PH (sens - TKI sensitive), BV (res - TKI-resistant), and KÖ (expressing BCR-ABL1^T315I^) - XTT assays upon exposure to increasing concentrations of crizotinib were performed. The Ph− PD-LTCs HP (Ph−) was used as a control. The means ± SD of three experiments each performed in triplicates are given. **f** Interaction of His-ABL with biotin-myristoyl-peptide (100%) and the displacement of the interaction by GNF2 and crizotinib

First, we studied the effect of crizotinib on BCR-ABL1 and BCR-ABL1^T315I^ in Ba/F3 cells showing that it inhibited not only the autophosphorylation of BCR-ABL1 and BCR-ABL1^T315I^ but also the substrate phosphorylation of STAT5 (Fig. 1a).

As constitutive kinase activity of BCR-ABL1 substitutes the survival signal of IL-3, the inhibitory effect of crizotinib prevented Ba/F3 cells expressing either BCR-ABL1 or BCR-ABL1^T315I^ from forming colonies in semi-solid media, whereas all other inhibitors (imatinib, nilotinib, and dasatinib and GNF-2) only suppressed BCR-ABL1, but not the “gatekeeper” mutant BCR-ABL1^T315I^ (Fig. 1b). In addition, crizotinib potently inhibited proliferation of all Ba/F3 expressing the resistance mutants BCR-ABL1^Y253F^, BCR-ABL1^T315I^, and BCR-ABL1^F317L^ in a dose-dependent manner with IC_50_ between 200 and 250 nM (Fig. 1c). No effect was seen on empty vector-transduced control cells in the presence of IL-3 at least until 1 μM (Fig. 1c). Crizotinib inhibited also the proliferation of Ph+ patient-derived cell lines such as BV-173 (lymphoblastic CML-BC) and SupB15 (Ph+ ALL) with an IC_50_ of about 50 nM and 150 nM, respectively, whereas no effect was seen on Ph− Jurkat control (Fig. 1d).

High-risk Ph+ leukemia is not fully represented by cell lines. Therefore, we examined the effects of crizotinib on Ph+ ALL patient-derived long-term cultures (PD-LTCs) with different response rates to TKI where a unique culture system allows to culture primary cells without genetic or immunophenotypical changes or any sign of senescence for at least 6 months [9]⁠. PH is fully responsive (sens) whereas BV exhibits an intrinsic resistance (res) to first-, second-, and third-generation TKIs. In contrast, KÖ cells harbor the BCR-ABL1^T315I^. HP, a Ph− PD-LTC, was used as a negative control. Crizotinib inhibited the proliferation of the PD-LTCs, with IC_50_ values between 50 and 100 nM and without affecting the proliferation of Ph− PD-LTC HP (Fig. 1e). Interestingly, both TKI-resistant BV and KÖ were inhibited by crizotinib at the same concentrations as the fully responsive PH. In contrast, these cells were irresponsive to dasatinib, nilotinib, and imatinib [10]⁠ and both need higher concentration of ponatinib or PF114 for the inhibition of BCR-ABL1^T315I^ as compared to native BCR-ABL1 [9]⁠. The fact that BV exhibited high sensitivity towards crizotinib not only is important for therapeutic reasons but also suggests that BCR-ABL1-independence can be excluded as a mechanism of resistance in these cells.

The fact that crizotinib exhibited nearly the same effect on native BCR-ABL1 as on BCR-ABL1^T315I^ raised the question of the modalities by which crizotinib inhibited ABL1 kinase activity. It has been shown that crizotinib only binds to the ATP-binding site of ABL1 [11]. Assuming that the changes of conformation of the ATP-binding site due to the presence of mutations were irrelevant for its activity, we hypothesized crizotinib acting as an allosteric inhibitor by changing the conformation of the MBP in BCR-ABL1. MBP is a hydrophobic pocket in the kinase domain that allosterically controls the activity of ABL1. It binds the myristoylated N-terminus (exon 1) in a process called “capping,” followed by conformational changes with the intra-molecular docking of the SH2 domain to TKD [12]⁠. This regulation is lost in t(9;22) by the substitution of ABL1’s exon 1 by BCR, allowing BCR-ABL1 to “escape” auto-inhibition. On the other hand, there is evidence that a variety of compounds binding at the ATP-binding site of protein kinases can allosterically affect interactions at distant regulatory sites [13]⁠. The ability to affect distant regulatory sites depends on the identity of the compound binding at the ATP-binding site. While some compounds either enhance or disrupt interactions, others may not produce any relevant effects at the regulatory sites [13]⁠. However, to the best of our knowledge, nothing is known about whether the conformation of the MBP of BCR-ABL1 can be modulated by compounds binding at the ATP-binding site. We tested the effect of crizotinib on the interaction of His-ABL1 with a myristoylated-peptide probe using AlphaScreen, an in vitro assay that is used to detect molecular interactions and is suitable to screen the effect of compounds. GNF2 displaced the interaction of ABL1 with the myristoylated probe (Fig. 1f), which was expected since it binds to the MBP and therefore competes with the myristoylated probe. Notably, crizotinib, which solely binds to the ATP-binding site on ABL1, also potently displaced the interaction of ABL1 with the myristoylated probe (Fig. 1f), indicating that the displacement was produced allosterically. Thus, our finding suggests that crizotinib acts on BCR-ABL1 inhibiting the kinase activity by a dual mechanism: (i) through the occupancy of the ATP-binding site; and (ii) affecting the conformation of the MBP as already shown for other kinases [14].

**Fig. 2:**
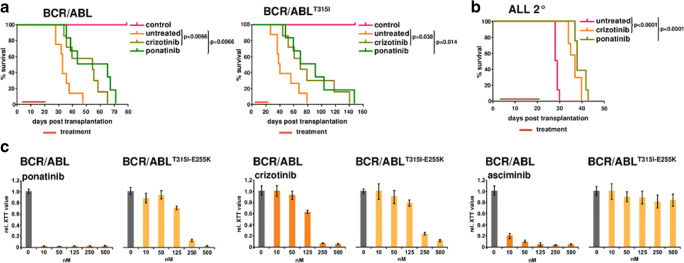
The efficacy of crizotinib in vivo in models of Ph+ leukemia. **a** For the induction of CML-like disease, sub-lethally irradiated C57BL/6N mice were transplanted intravenously with 1 × 10^5^ Sca1^+^-positive BM cells expressing BCR-ABL1 or BCR-ABL1^T315I^. Eight mice/group were treated orally either with crizotinib (100 mg/kg) or ponatinib (25 mg/kg) once daily for 20 days (treatment). For the design of in vivo experimentation, see the Supplementary Information. **b** Crizotinib prolongs the survival of mice with BCR-ABL1-derived ALL. 5 × 10^4^ spleen cells from ALL mice (frozen stock in liquid N_2_) were transplanted into sub-lethally (4.5 Gy) irradiated recipients. The mice were treated with crizotinib (100 mg/kg) or ponatinib (25 mg/kg) by gavage for 20 days. **c** Response of the compound mutation BCR-ABL1^T315I-E255K^ to crizotinib and ponatinib. The effect of crizotinib on the factor-independent growth of Ba/F3 expressing BCR-ABL1 or BCR-ABL1^T315I-E255K^ was performed on cells selected by IL-3 withdrawal. These cells were exposed to the indicated concentrations of PF-114, ponatinib, and asciminib. Cell proliferation and viability were assessed by XTT assays. The means ± SD of three experiments are given

In order to exclude that the higher dosages of crizotinib needed for the inhibition of BCR-ABL1 and its resistance mutants as compared to MET or ALK could limit its therapeutic potential in Ph+ leukemia, we evaluated the in vivo efficacy of crizotinib on native BCR-ABL1 and BCR-ABL1^T315I^ in a syngeneic mouse model for CML-like disease [9]⁠. All animal studies were conducted in accordance with national animal protection laws and were approved by the relevant monitoring institution (Regierungspräsidium Darmstadt - F 39/08). The mice were treated with crizotinib at the same dosage (100 mg/kg) already reported for the treatment of the ALK-positive Karpas 299 ALCL tumor xenograft model [15]⁠. Ponatinib (25 mg/kg) was used as a control. Both crizotinib and ponatinib extended median survival from 38 to 57 and 52 days (Fig. 2a). The same effect of crizotinib was observed when the CML-like disease was driven by BCR-ABL1^T315i^ (*p* = 0.038 for crizotinib and *p* = 0.014 for ponatinib) (Fig. 2a).

In order to study the effect of crizotinib in an advanced Ph+ leukemia, we treated syngeneic BCR-ABL1-driven ALL in the same way as CML-like disease with a very similar outcome (Fig. 2b) confirming that at the dosages used in the in vivo models for ALK-dependent tumors crizotinib performed comparably to ponatinib.

One future clinical challenge upon exposure to third-generation TKIs in high-risk Ph+ leukemia will be the emergence of “compound mutations,” multiple TKD mutations in one BCR-ABL1 allele. Mutation screens predict observations in patients BCR-ABL1^E255K-T315I^ to be resistant against ponatinib and PF-114 [9, 16]⁠. Here we show that crizotinib inhibited BCR-ABL1^E255K-T315I^ at the same concentration needed for native BCR-ABL1 in contrast to ponatinib which exhibited activity only starting from 125 nM which is far above its clinical feasible concentration.

In conclusion, crizotinib appears as a new subclass of inhibitor of BCR-ABL1, binding at the ATP-binding site and, allosterically, affecting the MBP regulatory site. The facts that it (i) is in an advanced stage of clinical development, (ii) acts as an allosteric inhibitor of BCR-ABL1, and (iii) represents a reserve in the case of resistance due to a compound mutation in BCR-ABL1 opens completely new therapeutic options and warrants consideration for clinical testing of crizotinib in Ph+ leukemia patients resistant to available TKI.

## Methods

### Compounds for in vitro and in vivo experiments

All compounds (imatinib, nilotinib, dasatinib, ponatinib, crizotinib, and asciminib) used in this study were purchased from Selleck Chemicals (Houston, TX, USA) and were diluted in DMSO to a 1000× stock solution, which then were diluted to 1× working concentrations for the experiments. For the in vivo experiments, the compounds were diluted to working concentration in 0.5% methyl-cellulose (Methocel® 65HG, Fluka, Darmstadt, Germany).

### BCR/ABL, its mutant, and ABL1 construct for proteins synthesis

p185^BCR/ABL^ constructs harboring point mutations related to TKI resistance (Y253F, E255K, T315I, F317L, and E255K-T315I) were cloned into the retroviral PINCO vector as shown previously [9]⁠⁠.

The catalytic domain of His-ABL1 was produced as previously described [17]⁠. In short, the catalytic domain of His-ABL1 (pET-28) was co-expressed with His-YopH (pCDFDuet) in BL21DE3 bacteria, purified through Ni-NTA chromatography and the catalytic domain of His-ABL1 further isolated through anion exchange chromatography.

### Cell lines and PD-LTCs

All of the cell lines used in this study were obtained from the German Collection of Microorganisms and Cell Cultures (DSMZ, Braunschweig, Germany). BV-173 and Jurkat cells were cultured in RPMI 1640 medium supplemented with 10% fetal calf serum (FCS) (Gibco/Invitrogen, Karlsruhe, Germany). SupB15 cells were cultured in RPMI 1640 containing 20% FCS. The packaging cell line Phoenix was cultured in DMSO with 10% FCS. Ba/F3 cells were grown in RPMI with 10% FCS supplemented with 10 ng of murine (m)IL-3 (PeproTech - Cell Concepts, Umkirch, Germany). Ph+ ALL PD-LTCs expressing BCR/ABL (PH, DW, KW, CM, and CR) or BCR/ABL-T315I (KÖ) were maintained in serum-free medium as described previously [18, 19]⁠.

### Western blotting

Western blot analyses were performed according to widely established protocols. The following antibodies were used: anti-ABL (α-ABL; Santa Cruz Biotechnology, Santa Cruz, CA, USA), anti-phosphorylated ABat Y245 (α-p-ABL-Y245), anti-STAT5 and anti-phosphorylated STAT5 (α-STAT5 and α-p-STAT5; Upstate-Biotechnology, Lake Placid, NY, USA). Blocking and antibody incubation were performed in 5% low-fat dry milk, followed by washing in Tris-buffered saline (TBS; 10 mM Tris-HCl, pH 8, 150 mM NaCl) containing 0.1% Tween-20 (TBS-T).

### Cytotoxicity, proliferation, and apoptosis

Cytotoxicity and proliferation were assessed using the XTT Proliferation Kit (Roche, Mannheim, Germany), according to the manufacturer’s instructions. Cell growth was assessed by dye exclusion using Trypan blue. Apoptosis was evaluated using 7-amino-actinomycin D (7-AAD) staining, as described previously [20]⁠. The IC_50_ was calculated using the Erithacus software (Erithacus Ltd., East Grinstead, UK).

### Isolation of Sca1^+^ hematopoietic stem and progenitor cells

Sca1^+^ cells were isolated from the BM of 8–12-week-old female C57BL/6N mice (Janvier, St. Berthevin, France) using the Sca1^+^ Enrichment Kit according to the manufacturer’s instructions (Miltenyi, Bergisch Gladbach, Germany).

### Retroviral infection

Ecotropic Phoenix packaging cells were transfected with the PINCO vectors. The retroviral supernatant was collected after 36 h. Sca1^+^ cells pre-stimulated for 2 days in DMEM, 10% FCS, mIL-3 (20 ng/mL), mIL-6 (20 ng/mL), and mSCF (100 ng/mL) (Cell Concepts) or Ba/F3 cells were plated onto RetroNectin-coated (Takara-Shuzo, Shiga, Japan) non-tissue culture 24-well plates and exposed to the retroviral supernatant for 3 h at 37 °C.

### Syngeneic transduction/transplantation model of CML and syngeneic BCR/ABL-induced ALL

All animal studies were conducted in accordance with national animal protection laws and were approved by the relevant monitoring institution (Regierungspräsidium Darmstadt - F 39/08). We used for these experiments a completely randomized design with independent experimental units. We defined cohorts with 7–8 mice/group [21]⁠. The animals were euthanized at the first appearance of morbidity [22]⁠, and disease was confirmed by post-mortem analysis of the spleen (data not shown).

For the transduction/transplantation model 8–12-week-old C57BL/6N mice, recipient mice were sub-lethally irradiated with 4.5 Gy. 10^5^ retrovirally transduced donor cells were inoculated via tail vein.

For the syngeneic ALL, cryo-preserved BM cells (4 × 10^4^) from C57BL/6N mice with BCR/ABL-driven ALL were injected via tail vein into sub-lethally irradiated (4.5 Gy) recipient.

### Colony-forming assay

A colony-forming assay in soft agar was performed as previously described [9]⁠. Briefly, cells (1 × 104) in 1 mL RPMI/10% FBS medium were diluted in 1 mL of 0.6% agar to give a final agar concentration of 0.3% agar. The cell-agar mixture was poured on top of a hardened agar base in wells of 12-well plates and allowed to solidify. Once the top layer solidified, 1 mL of medium containing different treatments was placed on top to keep the agar moist. The cells were grown at 37 °C in a 5% CO_2_-humidified atmosphere until colonies were visible (2–3 weeks). The plates were stained for 4 h with 5 mg/mL 3-(4,5-dimethylthiazol-2-yl)-2,5-diphenyltetrazolium bromide (MTT), and the dye was extracted with 1 mL solubilization buffer (20% sodium dodecyl sulfate (SDS), 50% *N*,*N*-dimethyl-formamide, 25 mM HCL) for 24 h. The optical density was measured at 570 nm wavelength with a reference wavelength of 630 nm.

### Alpha screen interaction displacement assay

The AlphaScreen assay was performed according to the manufacturer’s general protocol (Perkin Elmer, Rodgau, Germany). Reactions were performed in a 25 μL final volume in white 384-well microtiter plates (Greiner, Frickenhausen, Germany). His-tagged ABL1 (200 nM) and biotinylated myristoylated-peptide derived from the myristoylated N-terminus of ABL1 (myristoyl-GQQPGKVLGDQRRPSLK-Biotin) (GenScript Biotech, Piscataway, NJ, USA) (400 nM) were preincubated 30 m in 50 mM Tris-HCl (pH 7.4), 100 mM NaCl, 1 mM dithiothreitol, and 10 μM polyglycine in the presence of 1% DMSO (100% binding) or in the presence of different concentrations of compounds crizotinib or GNF2 diluted in DMSO. Subsequently, 5 μL of beads containing nickel chelate-coated acceptor beads and streptavidin-coated donor beads was added and incubated in the dark for 60′ at room temperature; the emission of light from the acceptor beads was measured in an EnVision reader (Perkin Elmer) and analyzed using the EnVision Spotfire® Analyst software (Perkin Elmer).
